# Screening common signaling pathways associated with drug resistance in non‐small cell lung cancer via gene expression profile analysis

**DOI:** 10.1002/cam4.2190

**Published:** 2019-04-25

**Authors:** Ting Sun, Qitai Zhao, Chaoqi Zhang, Ling Cao, Mengjia Song, Nomathamsanqa Resegofetse Maimela, Shasha Liu, Jinjin Wang, Qun Gao, Guohui Qin, Liping Wang, Yi Zhang

**Affiliations:** ^1^ Biotherapy Center The First Affiliated Hospital of Zhengzhou University Zhengzhou China; ^2^ Department of Respiratory medicine The First Affiliated Hospital of Zhengzhou University Zhengzhou China; ^3^ Cancer Center The First Affiliated Hospital of Zhengzhou University Zhengzhou China; ^4^ School of Life Sciences Zhengzhou University Zhengzhou China; ^5^ Engineering Key Laboratory for Cell Therapy of Henan Province Zhengzhou China

**Keywords:** common signaling pathways, drug resistance, non‐small cell lung cancer, transcriptome microarray data

## Abstract

Lung cancer is the leading cause of cancer‐related deaths worldwide. Although several therapeutic strategies have been employed to curb lung cancer, the survival rate is still poor owing to the development of drug resistance. The mechanisms underlying drug resistance development are incompletely understood. Here, we aimed to identify the common signaling pathways involved in drug resistance in non‐small cell lung cancer (NSCLC). Three published transcriptome microarray data were downloaded from the Gene Expression Omnibus (GEO) database comprising different drug‐resistant cell lines and their parental cell lines. Differentially expressed genes (DEGs) were identified and used to perform Gene Ontology (GO) enrichment analysis and Kyoto Encyclopedia of Genes and Genomes (KEGG) pathway analysis. An overlapping analysis was performed for KEGG pathways enriched from all the three datasets to identify the common signaling pathways. As a result, we found that metabolic pathways, ubiquitin‐mediated proteolysis, and mitogen‐activated protein kinase (MAPK) signaling were the most aberrantly expressed signaling pathways. The knockdown of nicotinamide phosphoribosyltransferase (*NAMPT*), the gene involved in metabolic pathways and known to be upregulated in drug‐resistant tumor cells, was shown to increase the apoptosis of cisplatin‐resistant A549 cells following cisplatin treatment. Thus, our results provide an in‐depth analysis of the signaling pathways that are commonly altered in drug‐resistant NSCLC cell lines and highlight the potential strategy that facilitates the development of interventions to interfere with upregulated signaling pathways as well as to boost downregulated signaling pathways in drug‐resistant tumors for the elimination of multiple resistance of NSCLC.

## 1 INTRODUCTION

Lung cancer is the most common and lethal malignancy, with a 5‐year survival rate of only 16%.^1^ Non‐small cell lung cancer (NSCLC) is the most common type of lung cancer that accounts for more than 80% of all lung cancer cases.^2^, ^3^ Chemotherapeutic drugs such as gemcitabine, taxane, and platin, alone or in combination, are the first line of treatment for patients with NSCLC. In addition, new therapeutic strategies have been applied for lung cancer treatment, including immunotherapy.^4^, ^5^, ^6^ Immunotherapy using antibodies that block immune checkpoints, programmed cell death 1 (PD‐1)/programmed cell death 1 ligand (PD‐L1), has shown impressive antitumor effects and clinical benefits for the treatment of NSCLC. The Food and Drug Administration (FDA) has approved anti‐PD‐1 antibodies, nivolumab, and pembrolizumab, for treatment of solid tumors including advanced NSCLC.^7^, ^8^, ^9^ The China Food and Drug Administration (CFDA) has recently approved nivolumab for the treatment of advanced NSCLC that lacks epidermal growth factor receptor (EGFR) mutations and anaplastic lymphoma kinase (ALK) mutations. However, most patients with late‐stage NSCLC show resistance to these drugs that has led to an increase in mortality rates.^10^ Drug resistance is a gene‐driven and pathway‐mediated process. Gene heterogeneities and mutations have been shown to play an important role in influencing drug efficacy and resistance in lung cancer; these phenomenon include mutations in tumor suppressor protein 53 (*TP53*),^6^, ^11^ kirsten rat sarcoma viral oncogene (*KRAS*),^12^, ^13^ PI3‐kinase subunit alpha (*PIK3CA*), PI3‐kinase subunit delta (*PIK3CD*),^14^, ^15^ and *EGFR*.^16^ In addition, noncoding RNAs (eg, microRNAs [miRNAs]) are known to alter drug resistance or sensitivity in lung cancer under chemotherapy treatment.^1^ These molecular mechanisms cause drug efflux, drug metabolism/inactivation, drug target alteration, DNA repair, or apoptosis deficiency. Over the past decades, therapies targeting either gene mutation or amplification have offered promising results, but the acquired resistance following treatment has demanded further investigations. It is acknowledged that genetic alterations in signaling pathways controlling apoptosis, cell cycle, and cell growth are common hallmarks of drug‐resistant tumors. Alterations in signaling pathways vary with different drug treatments. The drugs that target key molecules or block the downstream signaling pathways may cause feedback regulations and activate alternative signaling pathways to resist drug‐mediated destruction, as observed in tumor cells; this phenomenon has become a major obstacle for the elimination of drug resistance. Hence, it is essential to identify the common signaling pathways that mediate drug resistance.

Microarray technology has recently gained popularity for the investigation of gene alterations in tumorigenesis, metastasis, cancer recurrence, and drug resistance as well as to identify biomarkers for tumor diagnosis, prognosis, and therapy.^17^, ^18^, ^19^, ^20^, ^21^, ^22^ Through RNA‐sequencing analysis, many genes, RNAs, including messenger RNAs (mRNAs), long‐noncoding RNAs (lncRNAs), and miRNAs, and proteins have been reported to play a vital role in lung cancer initiation, progression, and recurrence. Larsen et al^23^ identified a distinct gene expression profile for recurrent lung squamous cell carcinoma through genome‐wide profiling. Sun et al^24^ reported the decrease in the expression of insulin‐like growth factor‐binding protein 3 (IGFBP3) in cisplatin‐resistant lung cancer cells, resulting in an increase in the activation of insulin‐like growth factor 1‐receptor (IGF‐1R) signaling and the subsequent resistance to cisplatin and radiation. Therefore, advances in RNA technology have made it possible to systematically study the genetic changes and commonly altered signaling pathways in drug‐resistant tumor cells.

**Figure 1:**
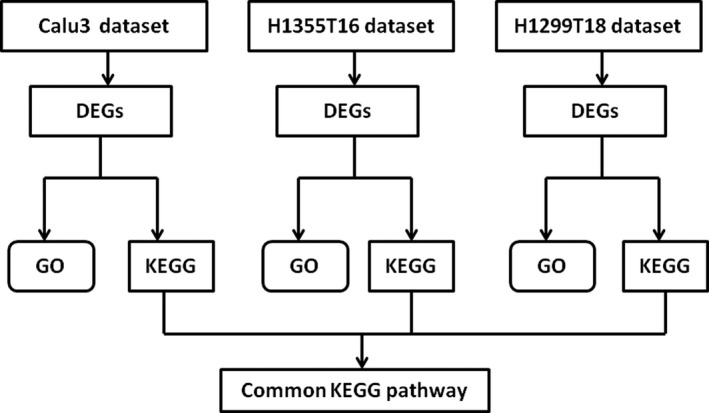
The multistep analyzed strategy used in this study

In this study, we screened the Gene Expression Omnibus (GEO) database and analyzed the selected three datasets with a multistep strategy (Figure 1). We first compared the differences at the gene level between drug‐resistant tumor cell lines and parental cell lines and identified several differentially expressed genes (DEGs). We performed Gene ontology (GO) and Kyoto Encyclopedia of Genes and Genomes (KEGG) pathway analyses of the DEGs for each dataset. An overlapping analysis of the KEGG pathways enriched from the three datasets was subsequently performed and 17 common pathways were identified. We detected the common signaling pathways associated with drug resistance and provided potential therapeutic options and possibilities to overcome drug resistance in patients with lung cancer.

## 2 MATERIALS AND METHODS

### 2.1 Microarray data

The transcriptome profiles of GSE6914 and GSE77209 were downloaded from GEO (https://www.ncbi.nlm.nih.gov/geo/) database. A total of 24 samples were used, including 10 drug‐resistant NSCLC cell lines and 14 parental cell lines. GSE6914 comprised one dataset, Calu3, containing four gemcitabine‐resistant Calu3 NSCLC cell lines and four parental cell lines. These eight samples were accessed using GPL96 Affymetrix Human Genome U133A Array. GSE77209 comprised two datasets, H1299T18 and H1355T16. The H1299T18 dataset had three taxane‐platin‐resistant H1299T NSCLC cell lines and five parental cell lines, while the H1355T16 dataset was composed of three taxane‐platin‐resistant H1355 NSCLC cell lines and five parental cell lines. These 16 samples were accessed using GPL10058 Illumina HumanHT‐12 v4.0 expression BeadChip.

### 2.2 Data preprocessing

Normalization is very crucial for the comparison of microarray datasets. We used the GEOquery package to download the raw probe‐level data (CEL. files) through the GEO database. The Z‐score method was used for the normalization of the three microarray data. Values of multiple probes that corresponded to the same gene were averaged as the final expression level for the specific gene.

### 2.3 Screening and clustering of the DEGs

The limma package was used for the identification of the DEGs between drug‐resistant cell lines and parental cell lines. The random variance model (RVM) *t* test was applied to filter the DEGs between the two groups, and the cut‐off value was set as |log (fold change)| >1.2 and false discovery rate (FDR) <0.05. Hierarchical clustering of the DEGs was based on the Euclidean distance, and was performed with EPCLUST.^25^, ^26^, ^27^ Venn diagram package was used to perform Venn analysis of the DEGs in three datasets. Unique DEGs were selected.

### 2.4 Enrichment analysis of unique DEGs

The GO analysis was used to analyze the biological functions of the genes, while KEGG pathway enrichment analysis was performed to investigate the signaling pathways that were related to the unique DEGs. The bioconductor package was used to perform GO and KEGG enrichment analyses. In particular, two‐sided Fisher's exact and chi‐squared tests were used to classify the GO category, FDR and q values were calculated to correct the *P*‐values.^28^, ^29^, ^30^, ^31^, ^32^ For the overlapping KEGG pathways, we overlapped the enriched KEGG pathways from the three datasets and identified the common KEGG pathways that were consistently altered in three datasets.

### 2.5 Cell culture

Human lung adenocarcinoma cancer cell line A549 (#SCSP‐503) and cisplatin‐resistant A549 (#ZQ0461) were purchased from the Chinese Academy of Sciences Cell Repertoire (Shanghai, China). All cells were cultured with 5% CO_2_ atmosphere at 37°C and maintained in Dulbecco's modified Eagle's medium (DMEM; Gibco, Grand Island, NY, #10569010) supplemented with 10% fetal bovine serum (HyClone, Logan, UT, #30068.03), 100 U/L of penicillin, and 100 μg/mL streptomycin (Thermo Fisher Scientific, Massachusetts, #15070063).

### 2.6 RNA extraction and real‐time polymerase chain reaction (RT‐PCR)

The total RNA from cisplatin‐resistant A549 cells was extracted with Trizol (Invitrogen Corporation, Carlsbad, CA, #A33250). NanoDrop 2000 (Thermo Scientific, Waltham, MA) was used to detect the concentration and purity of total RNA. The 1 μg of total RNA was reversely transcribed into complementary DNA (cDNA) with Prime Script RT reagent kit with genomic DNA eraser (TaKaRa, Tokyo, Japan, #RR037A). The primers used for cDNA preparation were oligo(dT). Real‐time quantitative PCR was performed on Agilent Mx3005P (Santa Clara, CA) consisting of specific primers and SYBR Premix Ex Taq II (TaKaRa, Tokyo, Japan, #RR8202). Primers used for this experiment are listed in Supplementary Table S10. All primers were purchased from Sangon Biotech (Shanghai, China), and the PCR program was as follows: 95°C for 30 s, 95°C for 5 s, and 60°C for 30 s. The range of CT values in this experiment was 21.25‐30.53. We used the 2^−ΔΔCt^ method for the analysis of the relative gene expression level. Each experiment was independently performed in triplicates, and glyceraldehyde 3‐phosphate dehydrogenase (*GAPDH*) was used for the normalization of data.

### 2.7 RNA interference

For transient expression knockdown, small‐interfering RNA (siRNA)‐NAMPT and negative control were designed and purchased from Gene Pharma Company (Shanghai, China); the siRNA sequences are shown in Supplementary Table S11. The siRNAs (si‐1‐NAMPT‐349, si‐2‐NAMPT‐1757, and si‐control‐NAMPT) were diluted in diethyl pyrocarbonate water at a final concentration of 20 μM according to the manufacturer's description. A549 and cisR‐A549 cells were plated in six‐well plates at a density of 5 × 10^5^ cells per well. After reaching approximately 60% confluency, the cells were transfected with the siRNA or negative control (2 μM/well) using Lipofectamine 3000 (Invitrogen, Carlsbad, CA, #L3000001) for 48 hours. After transfection, the cells were collected for RT‐PCR analysis or treated with cisplatin.

### 2.8 Flow cytometry for apoptosis analysis

After 24 hours of treatment with cisplatin, the supernatant was harvested and tumor cells were washed twice with ice‐cold phosphate‐buffered saline (PBS). The cells were suspended in Annexin‐V‐binding buffer (BioLegend, San Diego, CA, #422201) at a final concentration of 10^6^ cells/mL, and 100 μL of cell suspension was transferred into a 1.5 mL centrifuge tube and incubated with 5 μL of Alexa Fluor 647 Annexin‐V fluorescein isothiocyanate (FITC; BioLegend, San Diego, CA, #640906) and RNase (Thermo Fisher Scientific, USA, #AM2286) for 15 min at 4°C in the dark. After incubation, the samples were treated with propidium iodide (5 μL) (Sigma, Santa Clara, CA, USA, #P4170) and immediately analyzed with flow cytometry (BD, San Diego, CA, USA, FACSCanto II).

### 2.9 Western blot analysis

Radioimmunoprecipitation assay (RIPA) buffer (Solarbio, China, #R0010) mixed with protease and phosphatase inhibitors was used to isolate total proteins from cells. The protein concentration was measured using the bicinchoninic acid (BCA) Protein Assay Kit (Thermo Fisher Scientific, Massachusetts, #A53226), as per the manufacturer's instruction. Total protein was mixed with 5 × protein loading buffer (Beyotime, Shanghai, China, # P0280) at 1:4 ratio and boiled for 15 minutes. A total of 15 μg (10 μL volume) protein was added to 5% concentrated gel and 10% separation gel with 15 wells (Bio‐Rad, California, America, #1658000). After electrophoresis (80 V for 30 minutes and 120 V for 1.5 hours), the protein bands from sodium dodecyl sulfate polyacrylamide gel electrophoresis (SDS‐PAGE) gel were transferred onto nitrocellulose filter membranes (0.2 μm) (GE Healthcare Life science, Pittsburgh, Germany, #10600001) using the transfer slot (LiuYi Biological Co., Ltd., Beijing, China, #121‐4040) and SDS‐PAGE transfer buffer (Servicebio, Wuhan, China, #G2017) (200 mA for 1.5 hours). The membranes were stained with Ponceau S to investigate total protein level and washed thrice with TBST. The membranes were placed in a block buffer (TBST with 5% skim milk powder) for 1 hour at room temperature. After incubation, the membranes were washed thrice with TBST and incubated with 5% bovine serum albumin (BSA; Labio, Zhengzhou, China, #LB8020) containing anti‐total P44/42 mitogen‐activated protein kinase (MAPK; 1:1000 dilution; Cell Signaling Technology, #9102S), phosphorylated P44/42 MAPK(Thr202/Tyr204) (1:1000 dilution; Cell Signaling Technology, #4370T), and anti‐β‐actin (1:1000 dilution; Cell Signaling Technology, #3700S) overnight at 4°C. The membranes were washed thrice with TBST and incubated with a horseradish peroxidase (HRP)‐labeled goat anti‐rabbit IgG (1:3000 dilution, ZSGB‐BIO, Beijing, China, #ZB2301) for 1 hour at room temperature. Following incubation, the membranes were washed thrice with TBST and treated with an enhanced chemiluminescence (ECL) substrate (CWBIO, Beijing, China, #CW00495). FluorChem E System (Protein Simple, USA) was used to evaluate protein expression.

### 2.10 Statistical analysis

Data were analyzed with SPSS software (version 16.0; SPSS Inc, Chicago, IL) and GraphPad Prism 7 (version 5.0; San Diego, CA), and the results were presented as mean ± SD. The ratio data were subjected to log2 transformation and the proportion data were subjected to logit transformation. The two‐tailed unpaired *t* test was used to compare the difference in the relative gene expression and apoptosis ratios between experimental and control groups. The results were presented as histograms with overlaid dot plots; the whiskers represented error bars, and the upper box boundaries represented an average value. The dots represented the mean values of two technical repetitions. Each experiment has at least three biological replicates. *P*‐value less than 0.05 was considered statistically significant.

## 3 RESULTS

### 3.1 DEGs identified between drug‐resistant cell lines and control parental cell lines

**Figure 2:**
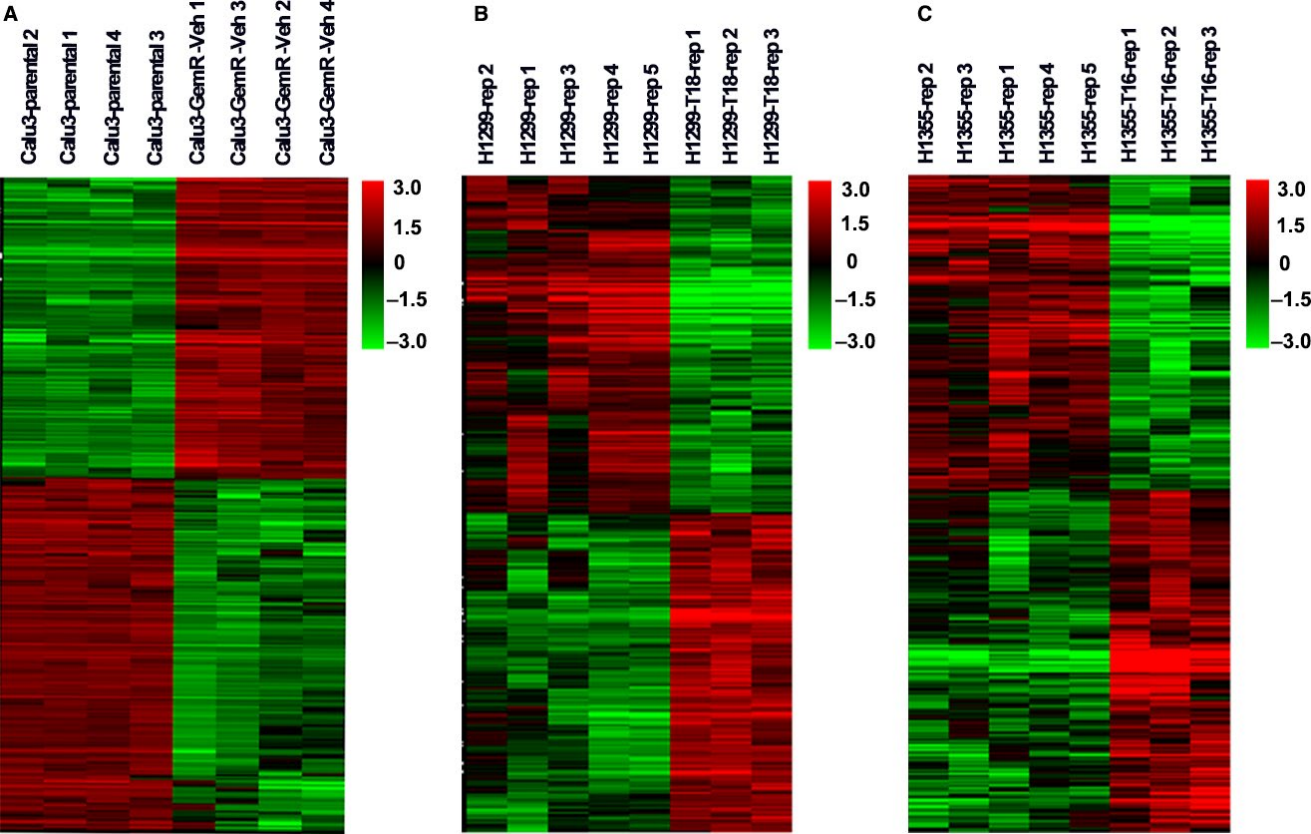
Cluster analysis of differentially expressed genes based on gene expression level in three datasets. A‐C, Represents the dataset of Calu3, H1299T18, and H1355T16

The transcriptome profiles of GSE6914 and GSE77209 were downloaded from the GEO database. The two profiles comprised 10 drug‐resistant cell lines and 14 paired parental cell lines. GSE6914 carried one dataset, Calu3, consisting of four gemcitabine‐resistant NSCLC cell lines, and four parental cell lines, while GSE77209 included two datasets, H1299T18 and H1355T16, comprising three taxane‐platin‐resistant NSCLC cell lines (H1299 or H1355) and five of their parental cell lines. The characteristics of the three individual datasets are shown in Table 1. We first identified the DEGs from the above three datasets and listed the top 10 DEGs (Supplementary Tables S1‐S3). We performed a two‐dimensional hierarchical clustering analysis of DEGs for each dataset. The results revealed the significant differences in the drug‐resistant cells as compared to their parental cells, as observed from the clustering of the DEGs. These observations indicate the high quality of these datasets and the reliability of the subsequent analysis (Figure 2).

**Table 1:** Characteristics of the three individual datasets

Dataset	GEO ID	Platform	Cell line	Samples
Calu3	GSE6914	GPL96 Affymetrix Human Genome U133A Array	Calu3	4 gemcitabine–resistant/4 parental cell lines
H1299T18	GSE77209	GPL10058 Illumina HumanHT‐12 V4.0 expression beadchip	H1299	3taxane+platin–resistant/5 parental cell lines
H1355T16	GSE77209	GPL10058 Illumina HumanHT‐12 V4.0 expression beadchip	H1355	3taxane+platin–resistant/5 parental cell lines

### 3.2 GO analysis of DEGs

**Figure 3:**
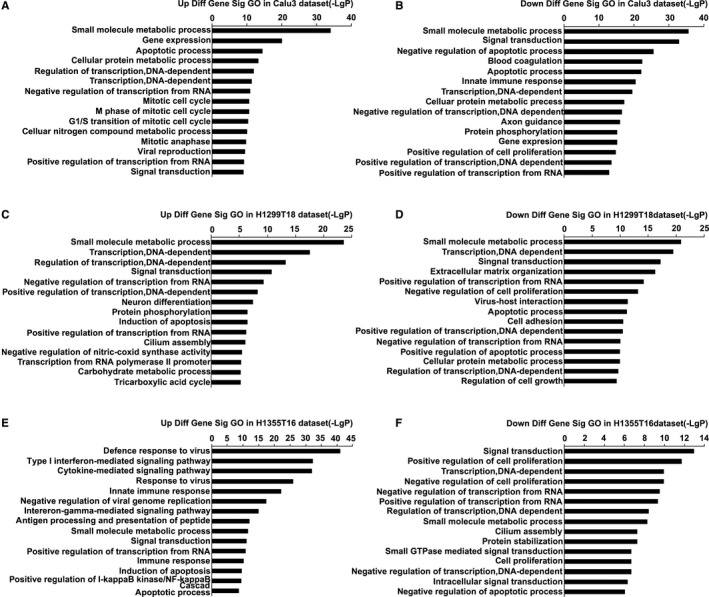
Top 15 Gene Ontology (GO) enrichment analysis of three datasets. A and B, Upregulated and downregulated of GO terms in the Calu3 dataset. C and D, Upregulated and downregulated GO terms in the H1299T18 dataset. E and F, Upregulated and downregulated of GO terms in the H1355T16 dataset

To investigate the functional role of the DEGs from the three independent datasets, we performed GO enrichment analysis. We observed 572 significantly enriched GO terms in Calu3 dataset, including 174 upregulated and 378 downregulated terms. In addition, we observed 307 GO terms in H1299T18 dataset, including 107 upregulated and 200 downregulated terms, and 343 GO terms in H1355T16 dataset, including 181 upregulated and 162 downregulated terms. The top 15 GO terms are shown in Figure 3, and the detailed information of the top 10 significantly upregulated or downregulated GO terms is listed in Supplementary Tables S4‐S6. The results showed the alterations in the small molecule metabolic process in drug‐resistant tumor cells.

### 3.3 KEGG pathway analysis of DEGs

**Figure 4:**
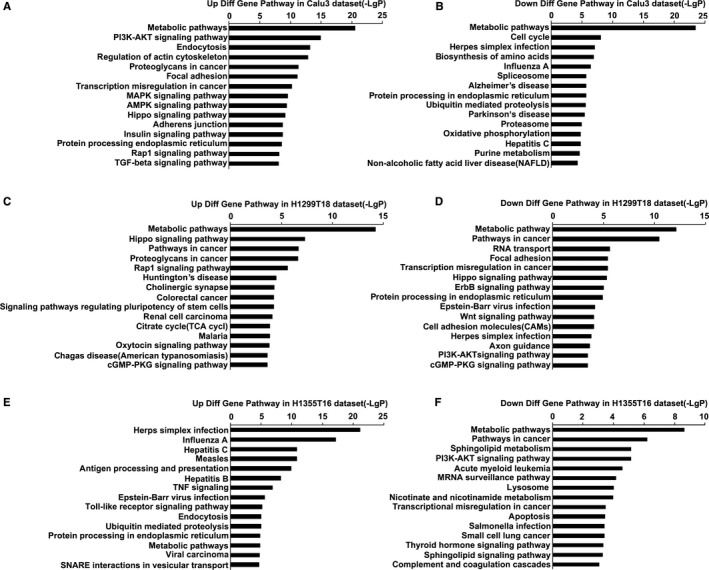
Top 15 Kyoto Encyclopedia of Genes and Genomes pathways (KEGG) analysis of three datasets. A and B, Upregulated and downregulated of KEGG pathways in the Calu3 dataset. C and D, Upregulated and downregulated of KEGG pathways in the H1299T18 dataset. E and F, Upregulated and downregulated of KEGG pathways in the H1355T16 dataset

To systematically understand the central pathways involved in drug resistance, we performed KEGG pathway analysis of the DEGs from the three datasets. The results revealed 180 significantly enriched KEGG pathways in Calu3 dataset, including 64 upregulated and 116 downregulated pathways, 89 KEGG pathways in the H1299T18 dataset, including 50 upregulated and 39 downregulated pathways, and 69 KEGG pathways in the H1355T16 dataset, including 47 upregulated and 22 downregulated pathways. The top 15 KEGG pathways are shown in Figure 4, and the detailed information of the top 10 significantly upregulated or downregulated KEGG pathways is listed in Supplementary Tables S7‐S9. A great difference was found in the enriched upregulated and downregulated pathways among the three datasets. Moreover, we found that the treatment of H1355 and H1299 with the same drugs taxane and platin resulted in variations in the alteration patterns, indicating that the cell lineage may affect the activation of signaling pathways. We also found that some signaling pathways were aberrantly altered in the three datasets (eg, metabolic pathways), indicating that these pathways were central for acquiring drug resistance.

### 3.4 Overlapping analysis of KEGG pathways

**Figure 5:**
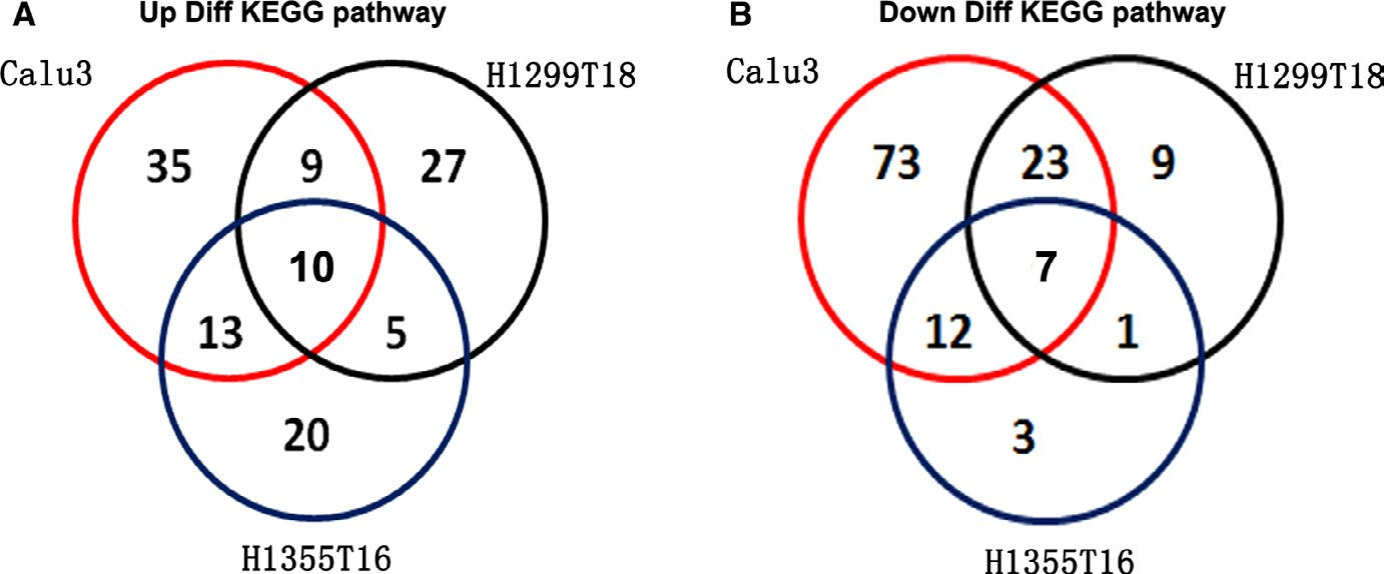
Venn diagram of the overlapping parts of KEGG pathways enriched in three datasets A, Identification of overlapping parts of three upregulated KEGG pathways. B, Identification of overlapping parts of three downregulated KEGG pathways

**Figure 6:**
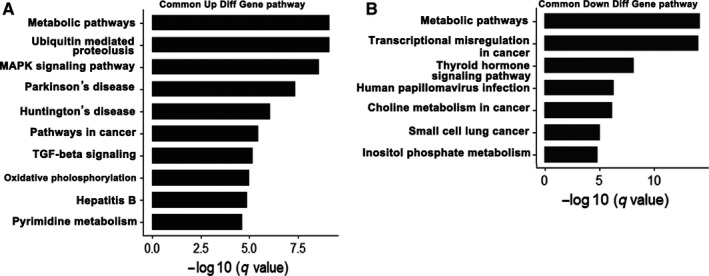
Common signaling pathways in three datasets. A, Upregulated common KEGG pathway in three datasets. B, Downregulated common KEGG pathways in three datasets

To further identify the KEGG pathways that were common in drug resistance, we performed an overlapping analysis of KEGG pathways that were enriched in all three datasets (Figure 5). We reported 17 overlapping KEGG pathways, including 10 upregulated and seven downregulated pathways (Figure 6), the detailed information is listed in Table 2. The results showed that metabolic pathways, upregulated ubiquitin‐mediated proteolysis, and MAPK signaling pathways were the major pathways mediating drug resistance, as evident from the most common significant alterations observed.

**Table 2:** Common Kyoto Encyclopedia of Genes and Genomes (KEGG) pathways in three datasets

KEGG ID	KEGG name	Enrichment	*P‐*value	False discovery rate(FDR)	Genes
Upregulated KEGG pathways					
01100	Metabolic pathways	5.183963	3.9E‐24	9.55E‐22	NAMPT, AASS, COQ7, CCD01, GNPDA1, CEPT1, FH, CDIPT, POLR3C, PMVK
04120	Ubiquitin‐mediated proteolysis	7.914154	2.65E‐06	7.2E‐05	CUL4B, ERCC8, UBE2J2, ANAPC5, UBE2D4, PML, DET1, UBA7, SKP1, SKP2
04010	MAPK signaling pathway	7.635487	3.68E‐06	9.01E‐05	AKT3, ATF2, DUSP10, GADD45A, TGF7, TGF9, FGFR2, FGFR4, HSPA1A, HSPB1
05012	Parkinson's disease	5.617819	5.69E‐05	0.000864	UBE2J2, COX8A, GNAl1, SLC25A6, APAF1, NDUFA2, NDUFA9, NDUFB2, SDHA, SDHC
05016	Huntington's disease	3.823513	9.69E‐05	0.001319	AP2S1, CLTB, COX8A, CREB1, APAF1, NDUFA2, NDUFA9, NDUFA10, NDUFB2, NDUFS2
05200	Pathways in cancer	4.677113	0.000118	0.001521	AKT3, CDK6, CDKN2A, LPAR1, F2R, FGF7, FGF9, FGFR2, FH, FN1
04350	TGF‐β signaling pathway	6.453804	0.004447	0.025337	DCN, BAMBI, ID4, SMAD3, SMAD5, PITX2, PPP2CA, BMP2, SKP1, BMP4
00190	Oxidative phosphorylation	5.556443	0.00678	0.043659	COX8A, ATP6V0E2, NDUFA9, NDUFB2, NDUFS3, NDUFV1, ATP6V1F, UQCRC1, SDHC, ATP6V1A
05161	Hepatitis B	4.455777	0.0098	0.041395	AKT3, CDK6, LAMTOR5, CREB1, ATF2, DDB2, DDX58, APAF1, IFNA10, IFNB1
00240	Pyrimidine metabolism	5.163043	0.011883	0.046958	POLR3C, CMPK2, TWISTNB, POLR2J2, NME7, ITPA, NME2, NT5C3A, POLR2K, POLR2J3
Downregulated KEGG pathways					
01100	Metabolic pathways	4.470121	3.1E‐21	7.63E‐19	FBP1, ALG3, B3GNT3, CEPT1, ACAA2, AGPAT2, SPTLC1, CEL, FTCD, MGLL
05202	Transcriptional misregulation in cancer	8.731303	6.8E‐11	2.39E‐09	CDK9, CEBPA, CEBPB, DUSP6, ETV5, HOXA9, HOXA10, ID2, IGFBP3, JUP
04919	Thyroid hormone signaling pathway	7.012028	2.52E‐05	0.000151	MED16, PLCD3, CTNNB1, AKT1, MTOR, ATP1B1, NOTCH1, NOTCH3, PLCD1,PLCD2
05165	Human papillomavirus infection	5.632562	1.60E‐08	0.003651	LAMC3, COL6A1, COL6A2, DVL1, DVL3, PPP2CB, TNC, RELA, RHEB, LAMA5
05231	Choline metabolism in cancer	4.856451	1.60E‐08	0.003565	CHKA, CHKB, RALGDS, RHEB, TSC1, PLPP3, PDGFC, PCYT1A, JUN, PIK3CB
05222	Small cell lung cancer	6.414103	0.001457	0.004371	POLR3C, CMPK2, TWISTNB, POLR2J2, NME7, ITPA, NME2, NT5C3A, POLR2K, POLR2J3
00562	Inositol phosphate metabolism	5.854564	0.03265	0.004523	MINPP1, SYNJ2, MTMR1, ITPKC, PI4K2A, PLCD1, PLCG2, INPP5K, PLCD3, ITPKB

### 3.5 Genes and common signaling pathways altered in cisplatin drug‐resistant tumor cells

**Figure 7:**
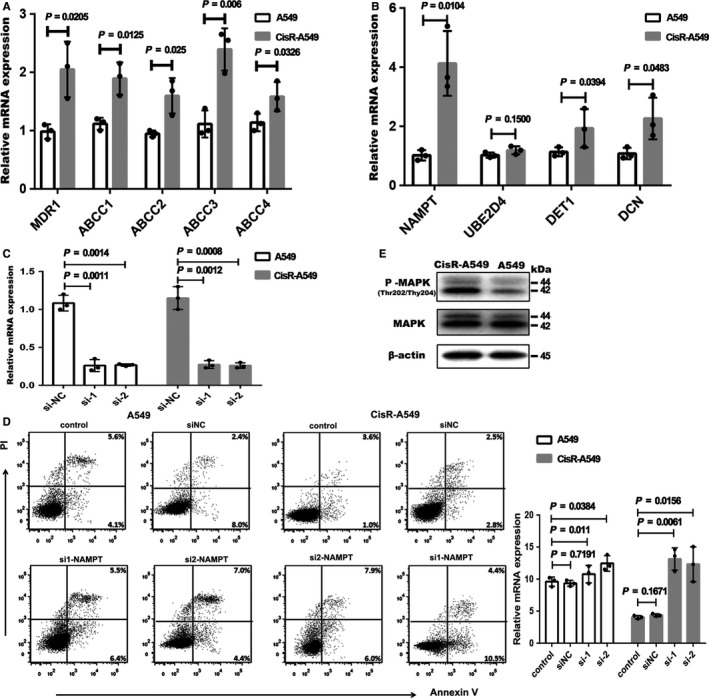
Validation of microarray data in cisplatin‐resistant A549 cell line. A, Drug‐resistant related genes mRNA expression in A549 and CisR‐A549 cells by RT‐PCR (CisR‐A549 represents cisplatin‐resistant A549). B, The identified genes mRNA expression in A549 and CisR‐A549 cells by RT‐PCR. C, The validation of knockdown efficiency by RT‐PCR. D, The apoptosis of A549 and CisR‐A549 cells upon cisplatin treatment (10 μg/mL) analyzed by flow cytometry (left panel). Statistical analysis of the rate of apoptosis cells upon cisplatin treatment (right panel). E, Representative western blot for total and phosphorylated P44/42 MAPK (Thr202/Tyr204) protein expression in A549 and CisR‐A549 cells. β‐actin was used as loading control. The number represents the protein size. Graphic display method refers to this articles.^62^, ^63^ The data in (A‐C) were made log2 transformation, and analyzed by unpaired *t* tests. The data in (D) were made logit transformation and analyzed by unpaired *t* tests. The dots represent the mean value of the two technical repetitions, results are representative of three independent experiments

We next used cisplatin‐resistant A549 tumor cells to validate the findings observed in our study. Some well‐known drug resistance‐related genes were detected with quantitative PCR, including multidrug resistance 1 (*MDR‐1*), ATP‐binding cassette subfamily C member 1 (*ABCC1*), *ABCC2*, *ABCC3*, and *ABCC4*. The results revealed that the expression of these genes was upregulated in cisplatin‐resistant tumor cells, suggesting that these cell lines are resistant to cisplatin (Figure 7A). We detected the mRNA expression levels of the genes that contributed to drug resistance identified in our study. As a result, we found that most of these genes were also upregulated in cisplatin‐resistant cells (Figure 7B). To further validate these results, we knockdown the expression of NAMPT with RNA interference, the efficiency of knockdown is shown in Figure 7C. The apoptosis of tumor cells was analyzed with flow cytometry, and the gating strategy is shown in Supplementary Figure S1. The knockdown of NAMPT expression dramatically increased the apoptosis of cisplatin‐resistant tumor cells following cisplatin treatment (Figure 7D), while minimum effects were observed on A549 cells. Western blot analysis showed that the expression of phosphorylated p44/42 MAPK(Thr202/Tyr204) was upregulated in cisplatin‐resistant tumor cells (Figure 7E) and unprocessed picture is shown in Supplementary Figure S2. These results suggest that the MAPK signaling pathways may be the common link in drug‐resistant tumor cells.

## 4 DISCUSSION

Somatic alterations in signaling pathways are common at varying frequencies and combinations in tumor cells and seem crucial in the development of resistance to various drugs. Therefore, the identification of the commonly altered signaling pathways in drug‐resistant tumor cells is essential for the development of effective therapeutic strategies.

In this study, we compared the gene expression profiles of 24 samples comprising gemcitabine‐resistant and taxane‐platin‐resistant NSCLC cell lines and their parental cell lines. We integrated three microarray datasets and identified the common signaling pathways associated with drug resistance. DEGs were identified for each dataset, and GO and KEGG enrichment pathway analysis for DEGs were performed to explore the molecular mechanisms underlying drug resistance development for each dataset. The functional enrichment analysis of GO terms and KEGG pathways showed striking differences between three drug‐resistant cell lines, indicating that the selective activation of signaling pathways is crucial for mediating drug resistance in tumor cells.

Drug resistance is a major obstacle observed during chemotherapy treatment, and different pathways are activated in the tumor cells in response to different drug treatments. Therefore, the identification of the common signaling pathways that are important to mediate drug resistance in NSCLC is desirable to eliminate drug resistance. We performed an overlapping analysis of three KEGG pathways for each dataset and found most significant alterations in metabolic pathways. Metabolic reprogramming is a hallmark of cancer development. Many studies have confirmed increased aerobic glycolysis, fatty acid synthesis, and glutamine metabolism to be associated with therapeutic resistance in cancer.^33^ In breast cancer, fatty acid synthase (FASN) induces docetaxel/trastuzumab/adriamycin resistance and lactate dehydrogenase A (LDHA) contributes to paclitaxel/trastuzumab resistance.^34^, ^35^ Aberrant metabolism has been thought to induce drug resistance in cancer cells; thus, the strategies targeting metabolism, for instance, glucose transporters (*GLUTs*), hexokinase (*HK*), pyruvate kinase M2 (*PKM2*), *LDHA*, pyruvate dehydrogenase kinase (*PDK*), and glutaminase (*GLS*), were shown to achieve a remarkable progress in reducing drug resistance in experimental and clinical studies.^36^, ^37^, ^38^, ^39^, ^40^, ^41^, ^42^, ^43^ Tavassoly et al^44^ reported different metabolic pathways in allopurinol‐sensitive and ‐resistant cell lines. Fatty acid catabolic process and triglyceride process were enriched in the resistant cells, while the pathways and processes related to oxidative stress were likely to be dominant in sensitive cells.

In this study, we found that NAMPT upregulation was the most common. NAMPT is a rate‐limiting enzyme in the salvage pathway for the biosynthesis of nicotinamide adenine dinucleotide (NAD^+^) from nicotinamide. NAMPT has been reported to activate MAPK signaling. As per our results, MAPK signaling was synergistically upregulated with NAMPT expression.^45^ In addition, NAMPT has also been associated with chronic inflammation in pancreatic cancer and is thought to contribute to drug resistance.^46^ The knockdown of NAMPT expression significantly increased the apoptosis of cisplatin‐resistant A549 tumor cells following cisplatin treatment. In contrast, the gluconeogenic enzyme fructose‐1,6‐bisphosphatase 1 (FBP1) is encoded by a well‐known tumor suppressor gene^47^ and known to be downregulated in drug‐resistant cell lines. These results indicate that the alternative metabolic reprogramming was essential in mediating resistance to drug killing. We similarly identified certain molecules such as alpha‐aminoadipic semialdehyde synthase (AASS) and coenzyme Q 7 homolog ubiquinone (COQ7) whose functions were unknown in tumor cells.

Ubiquitin‐mediated proteolysis pathway was the second most significantly upregulated pathway shared by the three datasets. Ubiquitin‐mediated proteolysis is involved in most cellular processes, including cell cycle, DNA repair, transcription, apoptosis, angiogenesis, protein synthesis, polyamine biosynthesis, and antigen presentation.^48^ The abnormal activation of ubiquitination has been linked to many diseases. Accumulating evidence has proved that the dysregulation in ubiquitin‐mediated proteolysis may play a crucial role in tumorigenesis and progression. The mutation in *Clb* gene, which is required for EGFR internalization and lysosomal degradation, results in the inhibition of the ubiquitin‐mediated degradation and has been linked to gastrointestinal tumor formation.^49^ However, in our results, we identified that Cullin 4B (*CUL4B*), a member of E3 ubiquitin ligases, was overexpressed in drug‐resistant cell lines. CUL4B promoted the degradation of P53 and inhibited the expression of phosphatase and tension homology (PTEN) through posttranscriptional modifications.^50^ In addition, recent studies have reported that CUL4B increases the expression of human EGFR2 (HER2) in gastric cancer cells to promote tumor invasion. Zhang et al showed that ubiquitination regulates the stability of *MDR1* gene product, P‐glycoprotein, and affects the functions of this membrane transporter, resulting in multidrug resistance. This phenomenon was confirmed by Liu et al These authors found that the seven‐in‐absentia homologue 1 (Siah1), an E3 ubiquitin ligase that regulates ubiquitination, was downregulated in P‐glycoprotein‐mediated multiple drug‐resistant cancer cells. Siah1 exhibits the function of decreasing P‐glycoprotein level, and low expression level of Siah1 was shown to induce multidrug resistance in cancer cells.^51^, ^52^ Therefore, the treatment of drug‐resistant cells with β‐elemene was shown to increase the expression of E3 ubiquitin ligases to enhance the efficacy of DOX treatment.^53^ This evidence, as well as our study, indicated the important role of ubiquitination in tumor progression.

MAPK signaling pathway is well‐known to participate in mediating drug resistance and is crucial for the damage‐induced DNA replication. Therefore, aberrant activation of MAPK signaling may prevent the death of cancer cells from drug treatment.^54^ Numerous studies have confirmed that MAPK signaling is involved in drug resistance induced by various chemotherapy drugs such as gemcitabine, platinum, 5‐fluorouracil, and tamoxifen.^55^, ^56^, ^57^, ^58^ Ercan et al^59^ revealed the amplification of *MAPK1* gene in EGFR kinase inhibitor‐resistant NSCLC cell lines. Inhibition of MAPK signaling using MEK inhibitor, CI‐1040, resulted in the restoration of sensitivity to WZ4002. Furthermore, MAPK feedback activation was another mechanism underlying drug resistance observed following EGFR inhibitor treatment. Dysregulation of MAPK signaling pathway was shown to correlate with the progression of pancreatic ductal adenocarcinoma. The blockade of MAPK signaling using FBP1‐derived small peptide could overcome the gemcitabine‐induced ERK activation and increase the efficacy of anticancer treatment.^60^, ^61^ In our study, upregulated MAPK signaling level was reported with a concomitant overexpression of activating transcription factor 2 (ATF2), an isoform of P38 MAPK family. Furthermore, dual‐specificity phosphatase 10 (DUSP10, MKP5) and downregulated FBP1 expression in drug‐resistant NSCLC tumor cell lines were observed among the three datasets. Fang *et al* also revealed the upregulated expression of MAPK signaling pathway in cisplatin‐resistant A549 cells, thereby supporting our analysis and validation (western blot analysis). These authors also found that the phosphoinositide 3‐kinase (PI3K)/protein kinase B (AKT) signaling pathway was upregulated in cisplatin‐resistant A549 cells. However, we failed to observe any consistent activation of PI3K/AKT signaling in the three datasets, as PI3K/AKT signaling was upregulated in gemcitabine‐resistant Calu3 cell line but downregulated in taxane‐platin‐resistant NSCLC cell line (H1299 or H1355).^30^ Janus kinase/signal transducer and activator of transcription (JAK‐STAT) signaling pathway have been reported to be upregulated in allopurinol‐resistant cell lines, but we failed to report similar observation in either taxane‐platin‐resistant cells or gemcitabine‐resistant cells. These differences may be related to the different killing mechanisms of the chemotherapy drugs.

To our knowledge, this is the first study to focus on the common signaling pathways involved in mediating drug resistance in NSCLC. Although many pathways identified in our study have already been known, our results prove that these pathways play an important role in mediating resistance to different drugs. In addition, we found that the genes in the same pathways may show different levels of alterations in response to different drugs, and revealed the functions of several genes involved in drug resistance. However, our study has a few limitations. For instance, only two types of drug‐resistant cell lines (gemcitabine and taxane‐platin) were used, only three cell lines were used, and the sample size was small. In addition, the backgrounds of tumor cell lines used were different, and our results lack the validation of mouse experiments and tumor specimen analysis. Further investigations in suitable mouse models and tumor samples from patients with NSCLC that are confirmed as drug resistant are warranted to validate the common pathways identified in this study.

In summary, our study provides a deep understanding of the signaling pathways that contribute to drug resistance in NSCLC and highlight some key molecules involved in these altered pathways. Notably, the most common signaling pathways identified in our study may facilitate the development of therapeutic interventions for drug‐resistant NSCLC.

## CONFLICT OF INTEREST

No potential conflicts of interest were disclosed.
