# Supplementary figures and images for: Screening common signaling pathways associated with drug resistance in non‐small cell lung cancer via gene expression profile analysis
Source: Cancer Med. 2019 Apr 25;8(6):3059–71. doi: 10.1002/cam4.2190 (PMC6558586; doi:10.1002/cam4.2190)

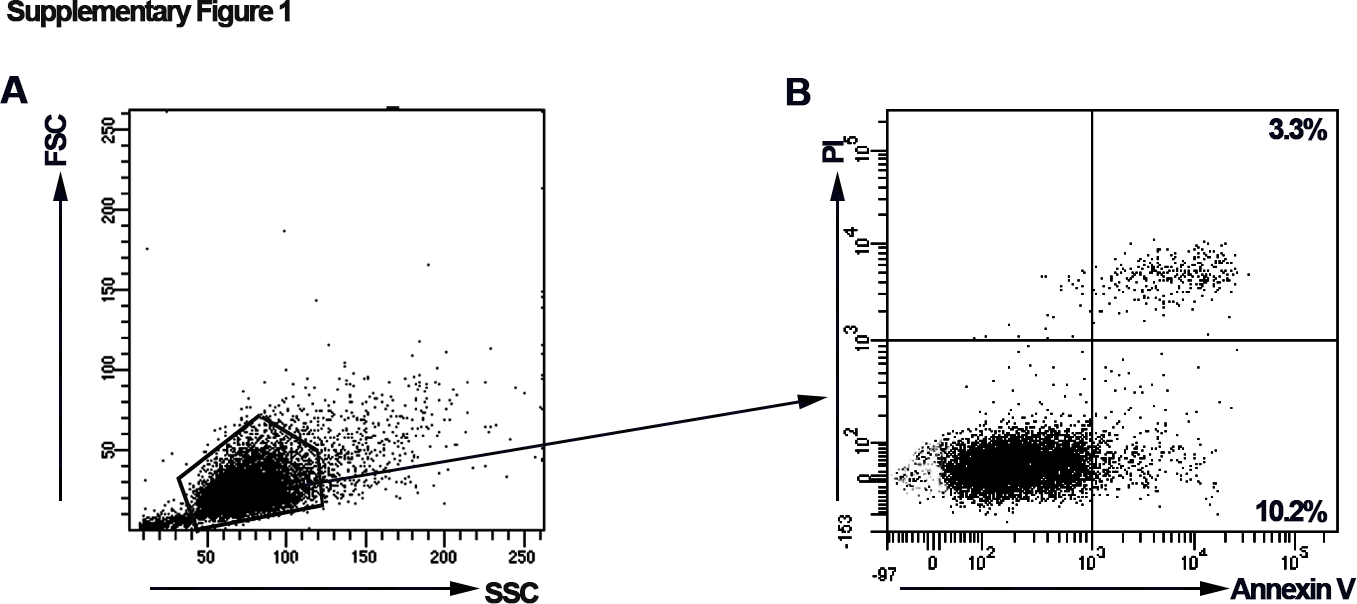

Supplement: Supplementary file 1 [file CAM4-8-3059-s001.tif]

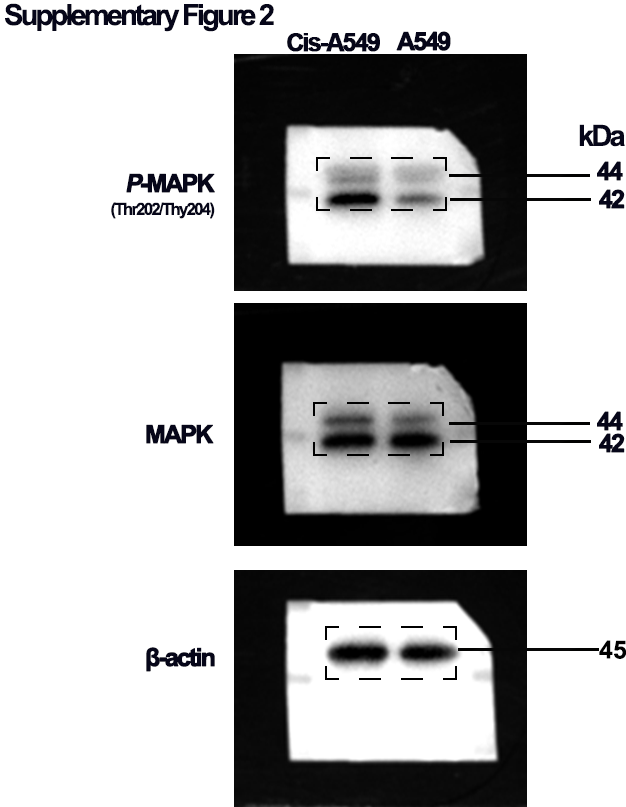

Supplement: Supplementary file 2 [file CAM4-8-3059-s002.tif]
