# Supplementary material for: Screening common signaling pathways associated with drug resistance in non‐small cell lung cancer via gene expression profile analysis
Source: Cancer Med. 2019 Apr 25;8(6):3059–71. doi: 10.1002/cam4.2190 (PMC6558586; doi:10.1002/cam4.2190)
Supplement: Supplementary file 3 [file CAM4-8-3059-s003.docx]

**Supplementary table 1: The top ten significant differentially expressed genes (DEGs) in Calu3 dataset.**

| Gene symbol | *P* value | Log FC | Function |
| --- | --- | --- | --- |
| CD37 | 0.0000001 | 3.31 | Transmembrane adhesive |
| CD81 | 0.0000001 | 3.51 | Transmembrane adhesive |
| DKK1 | 0.0000001 | 0.50 | Involved in cell biology |
| FGFR4 | 0.0000001 | 0.16 | Tumor promotion |
| MRPL17 | 0.0000001 | 6.55 | Encode mitochondrial |
| PSMC5 | 0.0000001 | 2.10 | recognize ubiquitinlabeled proteins, regulating  mammalian transcription |
| RRM1 | 0.0000001 | 8.34 | involved in DNA transcription and RNA translation |
| TRIM68 | 0.0000001 | 10.08 | Crosslink of protein |
| TPP1 | 1.3333E-07 | 4.83 | unknown |
| ACSL5 | 0.0000002 | 7.80 | Fatty acid metabolism |

**Supplementary table 2: The top ten significant DEGs in H1299T18 dataset.**

| Gene symbol | *P* value | Log FC | Function |
| --- | --- | --- | --- |
| C7 | 0.0000001 | 6.03 | Unknown |
| CGNL1 | 0.0000001 | 0.05 | cell-cell junction |
| H2AFB1 | 0.0000001 | 0.02 | Stable chromosome |
| H2AFB2 | 0.0000001 | 0.02 | Stable chromosome |
| KANK1 | 0.0000001 | 8.05 | Promote apoptosis |
| RFC3 | 0.0000001 | 3.51 | DNA replication and repair, Cell division and proliferation |
| TGFB2-AS1 | 0.0000001 | 3.6 | involved in early embryo development; Controlling the formation of cartilage, bone and sexual organs |
| CHST15 | 0.0000002 | 0.07 | regulates pathogenic mediators, tissue remodeling |
| IL15RA | 0.0000002 | 2.83 | osteoblast function and bone mineralization |
| TRPC3 | 0.0000002 | 3.91 | mediated calcium entry |

**Supplementary table 3: The top ten DEGs in H1355T16 dataset.**

| Gene symbol | *P* value | Log FC | Function |
| --- | --- | --- | --- |
| ABCB1 | 0.0000001 | 142.81 | wide-spectrum of ATP-dependent efflux pumps |
| HSH2D | 0.0000001 | 3.08 | Unknown |
| PLCL2 | 0.0000001 | 6.89 | Unknown |
| RUNDC3B | 0.0000002 | 64.87 | Unknown |
| DPYSL3 | 0.0000014 | 30.48 | cell-adhesions |
| SNRPD2P1 | 0.0000014 | 1.6 | Unknown |
| DYNLRB2 | 0.0000031 | 3.18 | transport stuff along the microtubule network |
| GAGE12C | 0.0000039 | 4.07 | Unknown |
| HCP5 | 0.000004 | 26.05 | long non-coding RNA |
| CATSPER1 | 0.0000056 | 3.68 | flagellum hyperactivation and, male fertility |

**Supplementary table 4. The top ten upregulated and downregulated Gene Ontology (GO) terms of Calu3 dataset.**

| **GO ID** | **GO name** | **enrichment** | **P -*value*** | **False discovery rate** (**FDR)** |
| --- | --- | --- | --- | --- |
| **Upregulated GO terms** | | | | |
| GO:0044281 | small molecule metabolic process | 5.966099397 | 1.05955E-34 | 1.84E-31 |
| GO:0010467 | gene expression | 6.65476113 | 9.22327E-21 | 8.01E-18 |
| GO:0006915 | apoptotic process | 5.636717192 | 3.63837E-15 | 2.10541E-12 |
| GO:0044267 | cellular protein metabolic process | 6.10542423 | 6.52262E-14 | 2.83E-11 |
| GO:0006355 | regulation of transcription, DNA-dependent | 3.658475593 | 1.0735E-12 | 3.72718E-10 |
| GO:0006351 | transcription, DNA-dependent | 3.085957021 | 4.68005E-12 | 1.3541E-09 |
| GO:0000122 | negative regulation of transcription from RNA polymerase II promoter | 5.538353122 | 1.35988E-11 | 3.37251E-09 |
| GO:0000278 | mitotic cell cycle | 6.571146245 | 2.35575E-11 | 5.04096E-09 |
| GO:0000087 | M phase of mitotic cell cycle | 9.309123847 | 2.6134E-11 | 5.04096E-09 |
| GO:0000082 | G1/S transition of mitotic cell cycle | 10.77058739 | 5.62595E-11 | 9.76666E-09 |
|  |  |  |  |  |
| **Downregulated GO terms** | | | | |
| GO:0044281 | small molecule metabolic process | 5.598419047 | 2.81248E-36 | 6.65152E-33 |
| GO:0007165 | signal transduction | 6.248042593 | 1.73776E-33 | 2.0549E-30 |
| GO:0043066 | negative regulation of apoptotic process | 8.357771261 | 3.10917E-26 | 2.45106E-23 |
| GO:0007596 | blood coagulation | 7.90842872 | 5.25915E-23 | 3.10947E-20 |
| GO:0006915 | apoptotic process | 6.466410181 | 1.0418E-22 | 4.92772E-20 |
| GO:0045087 | innate immune response | 6.803889601 | 4.08996E-21 | 1.61212E-18 |
| GO:0006351 | transcription, DNA-dependent | 3.623073256 | 3.24294E-20 | 1.0577E-14 |
| GO:0044267 | cellular protein metabolic process | 6.426558096 | 8.09109E-18 | 2.39193E-15 |
| GO:0045892 | negative regulation of transcription, DNA-dependent | 6.802401273 | 4.02508E-17 | 1.0577E-14 |
| GO:0007411 | axon guidance | 8.12048438 | 1.30156E-16 | 3.07818E-14 |

**Supplementary table 5. The top ten upregulated and downregulated GO terms of H1299T18 dataset.**

| **GO ID** | **GO name** | **enrichment** | **P -*value*** | **FDR** |
| --- | --- | --- | --- | --- |
| **Upregulated GO terms** | | | | |
| GO:0044281 | small molecule metabolic process | 4.228846813 | 2.7696E-24 | 5.48936E-21 |
| GO:0006351 | transcription, DNA-dependent | 3.241288327 | 3.18334E-18 | 3.15469E-15 |
| GO:0006355 | regulation of transcription, DNA-dependent | 3.330274617 | 5.91393E-14 | 3.90713E-11 |
| GO:0007165 | signal transduction | 3.372953864 | 1.79977E-11 | 8.91787E-09 |
| GO:0000122 | negative regulation of transcription from RNA polymerase II promoter | 4.343453717 | 5.73072E-10 | 2.27166E-07 |
| GO:0045893 | positive regulation of transcription, DNA-dependent | 4.1810639 | 5.77662E-09 | 1.90821E-06 |
| GO:0030182 | neuron differentiation | 10.33970976 | 4.02153E-08 | 1.13867E-05 |
| GO:0006468 | protein phosphorylation | 4.438455293 | 3.66366E-07 | 9.07671E-05 |
| GO:0006917 | induction of apoptosis | 6.073675665 | 4.12921E-07 | 9.09344E-05 |
| GO:0045944 | positive regulation of transcription from RNA polymerase II promoter | 3.122624212 | 6.94476E-07 | 0.000137645 |
|  |  |  |  |  |
| **Downregulated GO terms** | | | | |
| GO:0044281 | small molecule metabolic process | 3.887337782 | 1.59653E-21 | 3.47883E-18 |
| GO:0006351 | transcription, DNA-dependent | 3.308538997 | 3.58912E-20 | 3.91035E-17 |
| GO:0007165 | signal transduction | 4.057332397 | 6.84853E-18 | 4.97432E-15 |
| GO:0030198 | extracellular matrix organization | 9.239401496 | 6.28259E-17 | 3.42244E-14 |
| GO:0045944 | positive regulation of transcription from RNA polymerase II promoter | 4.426962255 | 7.32159E-15 | 3.19075E-12 |
| GO:0008285 | negative regulation of cell proliferation | 6.045117653 | 7.62785E-14 | 2.77018E-11 |
| GO:0019048 | virus-host interaction | 6.03769702 | 4.94194E-12 | 1.53836E-09 |
| GO:0006915 | apoptotic process | 4.107849642 | 6.75081E-12 | 1.83875E-09 |
| GO:0007155 | cell adhesion | 4.766854889 | 2.88276E-11 | 6.97949E-09 |
| GO:0045893 | positive regulation of transcription, DNA-dependent | 4.55962944 | 3.79522E-11 | 8.26979E-09 |

**Supplementary table 6. The top ten upregulated and downregulated GO terms of H1355T16 dataset.**

| **GO ID** | **GO name** | **enrichment** | **P -*value*** | **FDR** |
| --- | --- | --- | --- | --- |
| **Upregulated GO terms** | | | | |
| GO:0051607 | defense response to virus | 21.24766209 | 1.07793E-41 | 2.22053E-38 |
| GO:0060337 | type I interferon-mediated signaling pathway | 30.99846538 | 6.27698E-33 | 6.46529E-30 |
| GO:0019221 | cytokine-mediated signaling pathway | 13.81961762 | 1.25512E-32 | 8.61851E-30 |
| GO:0009615 | response to virus | 18.16979291 | 1.3804E-26 | 7.10906E-24 |
| GO:0045087 | innate immune response | 6.331081142 | 7.96197E-23 | 3.28033E-20 |
| GO:0045071 | negative regulation of viral genome replication | 33.70203523 | 4.62351E-18 | 1.5874E-15 |
| GO:0060333 | interferon-gamma-mediated signaling pathway | 18.36946096 | 1.31321E-15 | 3.8646E-13 |
| GO:0002474 | antigen processing and presentation of peptide antigen via MHC class I | 12.3094326 | 1.08014E-12 | 2.78135E-10 |
| GO:0044281 | small molecule metabolic process | 3.011318 | 3.37773E-12 | 7.73125E-10 |
| GO:0007165 | signal transduction | 3.332808755 | 8.85084E-12 | 1.82327E-09 |
|  |  |  |  |  |
| **Downregulated GO terms** | | | | |
| GO:0007165 | signal transduction | 3.70558944 | 1.14865E-13 | 2.35818E-10 |
| GO:0008284 | positive regulation of cell proliferation | 5.53239113 | 1.95269E-12 | 2.00444E-09 |
| GO:0006351 | transcription, DNA-dependent | 2.622467599 | 1.24777E-10 | 6.45457E-08 |
| GO:0008285 | negative regulation of cell proliferation | 5.444084807 | 1.25759E-10 | 6.45457E-08 |
| GO:0000122 | negative regulation of transcription from RNA polymerase II promoter | 4.467215628 | 3.03077E-10 | 1.24443E-07 |
| GO:0045944 | positive regulation of transcription from RNA polymerase II promoter | 3.785099924 | 4.82887E-10 | 1.65228E-07 |
| GO:0006355 | regulation of transcription, DNA-dependent | 2.802409453 | 3.94537E-09 | 1.15712E-06 |
| GO:0044281 | small molecule metabolic process | 2.74068197 | 5.13936E-09 | 1.31889E-06 |
| GO:0042384 | cilium assembly | 11.76921716 | 5.33831E-08 | 1.09595E-05 |
| GO:0050821 | protein stabilization | 11.76921716 | 5.33831E-08 | 1.09595E-05 |

**Supplementary table 7. The top ten upregulated and downregulated Kyoto Encyclopedia of Genes and Genomes(KEGG) pathways of Calu3 dataset.**

| **KEGG ID** | **KEGG name** | **enrichment** | **P -*value*** | **FDR** |
| --- | --- | --- | --- | --- |
| **Upregulated KEGG terms** | | | | |
| 01100 | Metabolic pathways | 4.470120772 | 3.10298E-21 | 7.63332E-19 |
| 04151 | PI3K-Akt signaling pathway | 7.461430575 | 1.16215E-15 | 1.42945E-13 |
| 04144 | Endocytosis | 8.19579895 | 7.20158E-14 | 5.90529E-12 |
| 04810 | Regulation of actin cytoskeleton | 9.021706361 | 1.48755E-13 | 9.14846E-12 |
| 05205 | Proteoglycans in cancer | 8.604798983 | 5.71043E-12 | 2.80953E-10 |
| 04510 | Focal adhesion | 8.438522674 | 8.08583E-12 | 3.31519E-10 |
| 05202 | Transcriptional misregulation in cancer | 8.731302937 | 6.80019E-11 | 2.38978E-09 |
| 04010 | MAPK signaling pathway | 6.850094877 | 3.09221E-10 | 9.50853E-09 |
| 04152 | AMPK signaling pathway | 10.3798127 | 4.10331E-10 | 1.12157E-08 |
| 04390 | Hippo signaling pathway | 8.954754922 | 7.36143E-10 | 1.12157E-08 |
|  |  |  |  |  |
| **Downregulated KEGG terms** | | | | |
| 01100 | Metabolic pathways | 5.183963428 | 3.90E-24 | 9.55E-22 |
| 04110 | Cell cycle | 10.4926367 | 8.41E-09 | 1.03E-06 |
| 05168 | Herpes simplex infection | 7.660385160 | 8.35E-08 | 6.82E-06 |
| 01230 | Biosynthesis of amino acids | 13.18669212 | 1.18E-07 | 7.19992E-06 |
| 05164 | Influenza A | 7.434782608 | 4.05E-07 | 1.98249E-05 |
| 03040 | Spliceosome | 8.091336794 | 2.16E-06 | 7.09425E-05 |
| 05010 | Alzheimer's disease | 7.099184782 | 2.18E-06 | 7.09425E-05 |
| 04141 | Protein processing in endoplasmic reticulum | 7.057177772 | 2.31649E-06 | 7.09425E-05 |
| 04120 | Ubiquitin mediated proteolysis | 7.914154237 | 2.64563E-06 | 7.20198E-05 |
| 05012 | Parkinson's disease | 7.635486834 | 3.67836E-06 | 9.01198E-05 |

**Supplementary table 8. The top ten upregulated and downregulated KEGG pathways of H1299T18 dataset.**

| **KEGG ID** | **KEGG name** | **enrichment** | **P -*value*** | **FDR** |
| --- | --- | --- | --- | --- |
| **Upregulated KEGG terms** | | | | |
| 01100 | Metabolic pathways | 3.519188088 | 6.06886E-15 | 1.47473E-12 |
| 04390 | Hippo signaling pathway | 7.177980331 | 5.00515E-08 | 6.08126E-06 |
| 05200 | Pathways in cancer | 4.176608203 | 2.19945E-07 | 1.50554E-05 |
| 05205 | Proteoglycans in cancer | 5.834318988 | 2.47826E-07 | 1.50554E-05 |
| 04015 | Rap1 signaling pathway | 5.238905076 | 2.51354E-06 | 0.000122158 |
| 05016 | Huntington's disease | 4.909292247 | 3.05996E-05 | 0.001239285 |
| 04725 | Cholinergic synapse | 6.401982457 | 5.12272E-05 | 0.001602681 |
| 05210 | Colorectal cancer | 8.914588476 | 5.46546E-05 | 0.001602681 |
| 04550 | Signaling pathways regulating pluripotency of stem cells | 5.560407299 | 5.93586E-05 | 0.001602681 |
| 05211 | Renal cell carcinoma | 8.374310386 | 8.28452E-05 | 0.002013138 |
|  |  |  |  |  |
| **Downregulated KEGG terms** | | | | |
| 01100 | Metabolic pathways | 3.205165773 | 8.23652E-13 | 2.01795E-10 |
| 05200 | Pathways in cancer | 5.07531549 | 3.65314E-11 | 4.4751E-09 |
| 03013 | RNA transport | 5.640332309 | 2.69898E-06 | 0.000207777 |
| 04510 | Focal adhesion | 5.047164697 | 3.87784E-06 | 0.000207777 |
| 05202 | Transcriptional misregulation in cancer | 5.419760654 | 4.24035E-06 | 0.000207777 |
| 04390 | Hippo signaling pathway | 5.815007935 | 5.226E-06 | 0.000213395 |
| 04012 | ErbB signaling pathway | 7.719924327 | 1.07679E-05 | 0.000376875 |
| 04141 | Protein processing in endoplasmic reticulum | 5.29888297 | 1.38306E-05 | 0.000423563 |
| 05169 | Epstein-Barr virus infection | 4.47755611 | 7.63661E-05 | 0.002078856 |
| 04310 | Wnt signaling pathway | 5.292619515 | 9.01966E-05 | 0.002135449 |

**Supplementary table 9. The top ten upregulated and downregulated KEGG of H1355T16 dataset.**

| **KEGG ID** | **KEGG name** | **enrichment** | **P -*value*** | **FDR** |
| --- | --- | --- | --- | --- |
| **Upregulated KEGG terms** | | | | |
| 05168 | Herpes simplex infection | 11.7616963 | 8.78308E-22 | 2.02889E-19 |
| 05164 | Influenza A | 10.66084788 | 7.99487E-18 | 9.23408E-16 |
| 05160 | Hepatitis C | 9.538653367 | 1.42319E-11 | 9.29681E-10 |
| 05162 | Measles | 9.467469386 | 1.60984E-11 | 9.29681E-10 |
| 04612 | Antigen processing and presentation | 12.59918386 | 1.3644E-10 | 6.30352E-09 |
| 05161 | Hepatitis B | 7.667048133 | 5.93783E-09 | 2.28607E-07 |
| 04668 | TNF signaling pathway | 8.141011109 | 1.31181E-07 | 4.32898E-06 |
| 05169 | Epstein-Barr virus infection | 5.223815461 | 2.56853E-06 | 7.41662E-05 |
| 04620 | Toll-like receptor signaling pathway | 7.040182562 | 7.07481E-06 | 0.000181587 |
| 04144 | Endocytosis | 4.338717161 | 1.06358E-05 | 0.000225352 |
|  |  |  |  |  |
| **Downregulated KEGG terms** | | | | |
| 01100 | Metabolic pathways | 2.895570769 | 2.2532E-09 | 5.18235E-07 |
| 05200 | Pathways in cancer | 4.09106289 | 6.34578E-07 | 7.29765E-05 |
| 00600 | Sphingolipid metabolism | 12.09474869 | 6.84518E-06 | 0.000412238 |
| 04151 | PI3K-Akt signaling pathway | 4.001533833 | 7.16935E-06 | 0.000412238 |
| 05221 | Acute myeloid leukemia | 9.972862958 | 2.58683E-05 | 0.001189941 |
| 03015 | mRNA surveillance pathway | 7.139129527 | 7.0455E-05 | 0.002700774 |
| 04142 | Lysosome | 5.942019393 | 9.37673E-05 | 0.002986056 |
| 00760 | Nicotinate and nicotinamide metabolism | 14.00131006 | 0.000103863 | 0.002986056 |
| 05202 | Transcriptional misregulation in cancer | 4.536737339 | 0.000337471 | 0.007406476 |
| 04210 | Apoptosis | 6.687684572 | 0.00035875 | 0.007406476 |

**Supplementary Table10. Primer sequences for qRT-PCR**

|  | **Forward** | **Reverse** |
| --- | --- | --- |
| MDR1 | 5’-GTCTTTGGTGCCATGGCCGT-3’ | 5’-ATGTCCGGTCGGGTGGGATA-3’ |
| ABCC1 | 5’-CTGACAAGCTAGACCATGAATGT-3’ | 5’-CCTTTGTCCAAGACGATCACCC-3’ |
| ABCC2 | 5’-GCCAGATTGGCCCAGCAAA-3’ | 5’-AATCTGACCACCGGCAGCCT-3’ |
| ABCC3 | 5’-GGGACCCTGCGCATGAACCTG-3’ | 5’-TAGGCAAGTCCAGCATCTCTGG-3’ |
| ABCC4 | 5'-GTTCTTCTGGTGGCTCAATCC3’ | 5'-GGCTTCTGTGCGTCATTCTC-3' |
| NAMPT | 5’-GGAAGCAAAGGAAGTGTACCGGGAAC-3’ | 5’-TCGTGCAGCTTATATTCAAGCCCGTC-3’ |
| UBE2D4 | 5’-GTGATGACTTGTTCCACTGGC-3’ | 5’-CCGCAGGATATCAAGGCAGA-3’ |
| DET1 | 5’-CGGGCAGAACAGGATGGTAG-3’ | 5’-ACAGCAATCACCTCTGTCGT-3’ |
| DCN | 5’-CAGCATTCCTCAAGGTCTTCCT-3’ | 5’-GAGAGCCATTGTCAACAGCA-3’ |
| GAPDH | 5’-AGGAGCGAGATCCCTCCAAAAT-3’ | 5’-GGCTGTTGTCATACTTCTCATGG-3’ |

**Supplementary Table 11. Sequences of the siRNAs targetingNAMPT.**

| Si-Control | 5’-UUCUCCGAACGUGUCACGUTT-3’ |
| --- | --- |
| Si1-NAMPT | 5’-CCACCGACUCCUACAAGGUUACUCA-3’ |
| Si2-NAMPT | 5’-UAUUGAACUGGAAGCAGCACAUCAU-3’ |
